# Standardized data to support conservation prioritization for sharks and batoids (Elasmobranchii)

**DOI:** 10.1016/j.dib.2020.106337

**Published:** 2020-09-24

**Authors:** Rikke Oegelund Nielsen, Rita da Silva, Jacqueline Juergens, Johanna Staerk, Line Lindholm Sørensen, John Jackson, Simeon Quirinus Smeele, Dalia A. Conde

**Affiliations:** a Department of Biology, University of Southern Denmark, Campusvej 55, 5230 Odense M, Denmark; b Species360 Conservation Science Alliance, 7900 International Drive, Suite 1040, Bloomington, MN 55425, USA; c Interdisciplinary Centre on Population Dynamics, University of Southern Denmark, 5230 Odense M, Denmark; d Biological Faculty, University of Hamburg, Martin-Luther-King-Platz 3, 20146 Hamburg, Germany

**Keywords:** Aquatic management, CITES, Threatened species, International treaties, Life history traits, Captive husbandry, Aquariums, rays

## Abstract

We collated and synthesized information on 1,226 Elasmobranch species (i.e., sharks, rays, and skates) globally from a wide range of sources. We obtained curated and standardized data from online databases, legal documents, press releases, and websites. All data were standardized according to the taxonomic nomenclature described in the Catalogue of Life. We grouped data into five categories: 1) biological information, 2) conservation status, 3) management opportunities, 4) use, and 5) inclusion in international conventions and treaties. For species biological information, we included migration, habitat, species characteristics such as length & body weight, their threat to humans, life-history trait data availability from FishBase, whether the species was listed on the Global Register of Migratory Species, the presence of occurrence data from the Global Biodiversity Information Facility (GBIF), information on genomics from GenBank, and species evolutionary distinctiveness scores. For conservation status, we recorded threat status from the International Union for Conservation of Nature Red List of Threatened Species™ and inclusion in the Alliance for Zero Extinction (AZE). For management opportunities, we identified species under human care in zoos and aquariums in the Species360 network, species under management in studbooks from the European Association of Zoos and Aquaria (EAZA), the American Association for Zoos and Aquariums (AZA), and the Zoo and Aquarium Association Australasia (ZAA), as well as data on recovery, management, and action plans at the class, family, and species levels. For use, we collated species-level data on international trade levels from the CITES (the Convention on International Trade in Endangered Species of Wild Fauna and Flora) Trade Database, as used in aquaculture, as bait, and as gamefish, recording the purpose of the trade according to the IUCN Red List and the global catches reported to the FAO (Food and Agriculture Organization of the United Nations). Finally, we collated information from seven international conventions and treaties: CITES, UNCLOS (the United Nations Convention for the Law of the Sea), CMS (the Convention on the Conservation of Migratory Species of Wild Animals), Shark MoU (the Memorandum of Understanding on the Conservation of Migratory Sharks), BERN (the Convention on the Conservation of European Wildlife and Natural Habitats), OSPAR (Protecting and conserving the North-East Atlantic and its resources), and the Barcelona Convention for the Protection of the Marine Environment and the Coastal Region of the Mediterranean. Our data are comparable across databases and will assist further research on in-situ and ex-situ population management for sharks and batoids. Our data can be of use to international policy makers, aquarium curators, management authorities, conservation practitioners, and scientists interested in prioritizing Elasmobranchs for conservation.

## Specifications Table

**Table:** 

Subject	Management, Monitoring, Policy and Law
Specific subject area	Species-level data on Elasmobranchs to support conservation prioritization
Type of data	Table Figure Chart
How data were acquired	Open-access online databases, legal documents, press releases, and websites.
Data format	Raw, Analyzed and Filtered
Parameters for data collection	We collated data on all Elasmobranchs (i.e., sharks and batoids) described in the Catalogue of Life (1,226 species). We collated data into five categories: 1) biological information, 2) conservation status, 3) management opportunities, 4) use, and 5) inclusion in international conventions and treaties.
Description of data collection	Most datasets were downloaded from online sources in 2019 and 2020 (links provided in Table 1). For species not yet included in online databases, we used press releases. We standardized species names according to the Catalogue of Life and completed all data collation, processing, and analysis in the open-source software R.
Data source location	Global.
Data accessibility	With the article

## Value of the Data

• This is a comprehensive collection of datasets on Elasmobranch species (i.e., sharks and batoids), comprising their biological traits, occurrence, migration, body characteristics, habitat, genomics, conservation status, trade patterns, threat to humans, fishery catches, catching methods, and inclusion in seven international conventions, treaties, and prioritization schemes.
• These data will provide key foundational information for the conservation of Elasmobranchs globally.
• These data can be re-used to support decision making for ex-situ and in-situ conservation, for example by aquariums (e.g., collection planning and captive management), conservation organizations, governments, and global conventions and treaties.
• The dataset identifies salient knowledge gaps and conservation and research opportunities for the Elasmobranchs.

## 1 Data Description

The presented data cover 1,226 Elasmobranchs (i.e., sharks, rays, and skates) described in the Catalogue of Life [1]. We used the Catalogue of Life as a standardized taxonomy to access information across a range of open-source data repositories (Table 1). The supplementary dataset Supplementary File S3 is a list of species’ scientific names used for the following datasets: Catalogue of Life (CoL), FishBase, IUCN Red List, CITES, and ZIMS (Species360 Zoological Information Management System). This data file can be used as a taxonomic translation table among datasets. We further cross-matched the species taxonomy from CoL with the taxonomy from the World Register of Marine Species (WoRMS) which is available from the Supplementary File S1. The Supplementary File S1 shows merged data for the following five categories: 1) biological information, 2) conservation status, 3) management opportunities, 4) use, and 5) inclusion in international conventions and treaties. The Supplementary File S2includes explanations of all data, variables, their sources and original column names.

**Table 1:** The number of Elasmobranch species assessed in each dataset, with data types and sources. We also quantify overlap with species described in the Catalogue of Life (CoL). Comparing the second and third columns shows how many species will be lost when merging datasets with the Catalogue of Life taxonomy. Further data sources for management opportunities are described in Table 7. Note: For the data from the IUCN Red List Advanced search we show the number of different species in each of the corresponding data files under the Title column. Additionally, please note that some databases contain data used in more than one category even though the database is only listed under one of the categories in this table (refer to the category column in the Supplementary File S1).

Dataset	Number of species	Number of species also in CoL	Date of access or version	Title	Link
Catalogue of Life	1,226	-	May 2019	Annual checklist of the world's known species	http://www.catalogueoflife.org/annual-checklist/2019/

1) Biological information					

FishBase	1,233	1,221	March 2020	Information Gaps	https://www.fishbase.se/tools/InformationGaps/menu.php
FishBase data	1,234	1,226	August 2020	R Interface to 'FishBase'	http://www.fishbase.org
Global Register of Migratory Species (GROMS)	66	66	March 2020	Summarizing knowledge about migratory species for conservation	http://www.groms.de/
Global Biodiversity Information Facility (GBIF)	1,017	946	April 2020	Occurrence records	https://www.gbif.org/
GenBank	736	724	August 2019	GenBank	https://www.ncbi.nlm.nih.gov/genbank/
Vertebrate Genome Project (VGP)	13	13	February 2020	VGP public ordinal list	https://vertebrategenomesproject.org/
Evolutionary Distinctiveness (ED)	1,192	1,113	February 2020	EDGE of Existence	https://www.edgeofexistence.org/edge-lists/
IUCN Red List Advanced Search	1,077 / 977 and 1,082	1,066 / 970 and 1,070	August 2020 (v. 2020–2)	“countries” / “threats” and “all_other_fields”	https://www.iucnredlist.org/

2) Conservation status					

IUCN Red List	1,077	1,068	March 2020 (v. 2020–1)	IUCN Red List of Threatened Species™	https://www.iucnredlist.org/
Alliance for Zero Extinction (AZE)	2	2	October 2019	2018 global AZE map	https://zeroextinction.org/site-identification/2018-global-aze-map/

3) Management opportunities					

Species360 Zoological Information Management System (ZIMS)	159	151	May 2019	ZIMS species holdings	https://www.species360.org/

4) Use					

The Convention on International Trade in Endangered Species of Wild Fauna and Flora (CITES)	70	70	January 2020	UNEP-WCMC CITES Trade Database	https://trade.cites.org/
Food and Agriculture Organization of the United Nations (FAO)	142	142	March 2020	Global capture production 1950–2017	http://www.fao.org/fishery/statistics/global-capture-production/query/en
IUCN Red List Advanced Search	479	478	August 2020 (v. 2020–2)	“usetrade”	https://www.iucnredlist.org/
5) Conventions and treaties					

The Convention on International Trade in Endangered Species of Wild Fauna and Flora (CITES)	70	70	March 2020	Checklist of CITES Species and Species+	http://checklist.cites.org/#/en and https://speciesplus.net/
The United Nations Convention on the Law of the Sea (UNCLOS)	80	80	Original law from 1982 Article 64: Highly migratory species.	Annex I: Highly migratory species	https://www.un.org/depts/los/convention_agreements/texts/unclos/unclos_e.pdf
The Convention on the Conservation of Migratory Species of Wild Animals (CMS)	37	37	November 2019 (added new species in February 2020[2])	CMS species	https://www.cms.int/en/species
The Memorandum of Understanding on the Conservation of Migratory Sharks (Shark MoU)	37	37	November 2019	CMS instrument: Sharks	https://www.cms.int/en/species
The Convention on the Conservation of European Wildlife and Natural Habitats (BERN)	8	8	1998 version, ETS No. 104	Appendix II: Strictly protected fauna species Appendix III: Protected fauna species	https://www.coe.int/en/web/conventions/full-list/-/conventions/treaty/104
Protecting and conserving the North-East Atlantic and its resources (OSPAR)	11	11	From agreement 2008–6	List of threatened and/or declining species and habitats	https://www.ospar.org/work-areas/bdc/species-habitats/list-of-threatened-declining-species-habitats
Convention for the Protection of the Marine Environment and the Coastal Region of the Mediterranean (Barcelona Convention)	33	33	February 2012 version December 2017 version	Annex II: List of endangered or threatened species Annex III: List of species whose exploitation is regulated	http://www.rac-spa.org/sites/default/files/annex/annex_2_en_20182.pdf. http://www.rac-spa.org/sites/default/files/annex/annex_3_en_2013.pdf.

### 1.1 Biological information

This category summarizes information available in open-source repositories on species biological traits, migratory status, occurrence data, species characteristics, habitat, genomics, and evolutionary distinctiveness. We merged data from FishBase, the Global Register of Migratory Species (GROMS), the Global Biodiversity Information Facility (GBIF), the IUCN Red List, GenBank, the Vertebrate Genome Project (VGP), and evolutionary distinctiveness scores from the EDGE of Existence database [3]. We included two types of data retrieved from Fishbase, 1) whether or not biological traits are available from FishBase [4] in the following categories: reproduction, photo identification, ecology, food, maturity, maximum size, spawning, length and weight, eggs, diet, growth, genetics, predators, mortality and hatchlings (Table 2). We assumed that data existed for a given category if at least one of the parameters was available for that species. 2) Data on common names, habitat, body shape, water zone, migration, depth ranges and common depth ranges, longevity, price category, length, body weight, resilience to fishery, their electrogenic abilities, threat to humans, vulnerability to fishery and trophic level. More details on each information type are available in the Supplementary File S2 and Table 5. For references on these, we refer to Fishbase.org. Additionally we included data on country distribution, threats, stressors, generation length, and migration patterns from the IUCN Red List of Threatened Species™ Advanced Search.

**Table 2:** Summary of available biological data for 537 shark and 689 batoid species on FishBase Information Gaps database [5].

	Number of species with data (%)		
Category	Sharks	Batoids	Parameters [5]
Reproduction	427 (79.5%)	443 (64.3%)	Reproductive mode, fertilization strategy, mating system, reproductive guild, spawning frequency
Photos	300 (55.9%)	391 (56.7%)	Color pictures
Ecology	289 (53.8%)	266 (38.6%)	Information on environment, feeding habit, and trophic level
Food	224 (41.7%)	220 (31.9%)	Lists of organisms found in the stomach or otherwise known to be consumed
Maturity	252 (46.9%)	178 (25.8%)	Length and age at sexual maturity
Maximum size	188 (35.0%)	155 (22.5%)	Maximum reported length
Spawning	198 (36.9%)	45 (6.5%)	Spawning season, sex ratio, absolute and relative fecundity, temperature at spawning, fecundity-length relationship, daily spawning frequency, gestation period and length of offspring for live-bearing species
Length and weight	120 (22.3%)	116 (16.8%)	Parameters to estimate wet weight at a given length
Eggs	11 (2.0%)	224 (32.5%)	Egg characteristics and environmental parameters associated with the occurrence of eggs
Diet	162 (30.2%)	50 (7.3%)	Percentages of different food items reported from stomach contents
Growth	79 (14.7%)	61 (8.9%)	Growth parameters related to changes in body mass over time
Genetics	43 (8.0%)	39 (5.7%)	Chromosome information or DNA studies
Predator	32 (6.0%)	25 (3.6%)	Documented instances of a predator (any taxa) having consumed the fish of concern
Mortality	24 (4.5%)	6 (0.9%)	Rate of death from various causes
Hatchling	7 (1.3%)	4 (0.6%)	Detailed information on morphological characteristics, meristic characters in different life stages, length at hatching, larval duration, length at first feeding and water parameters

**Fig. 1:**
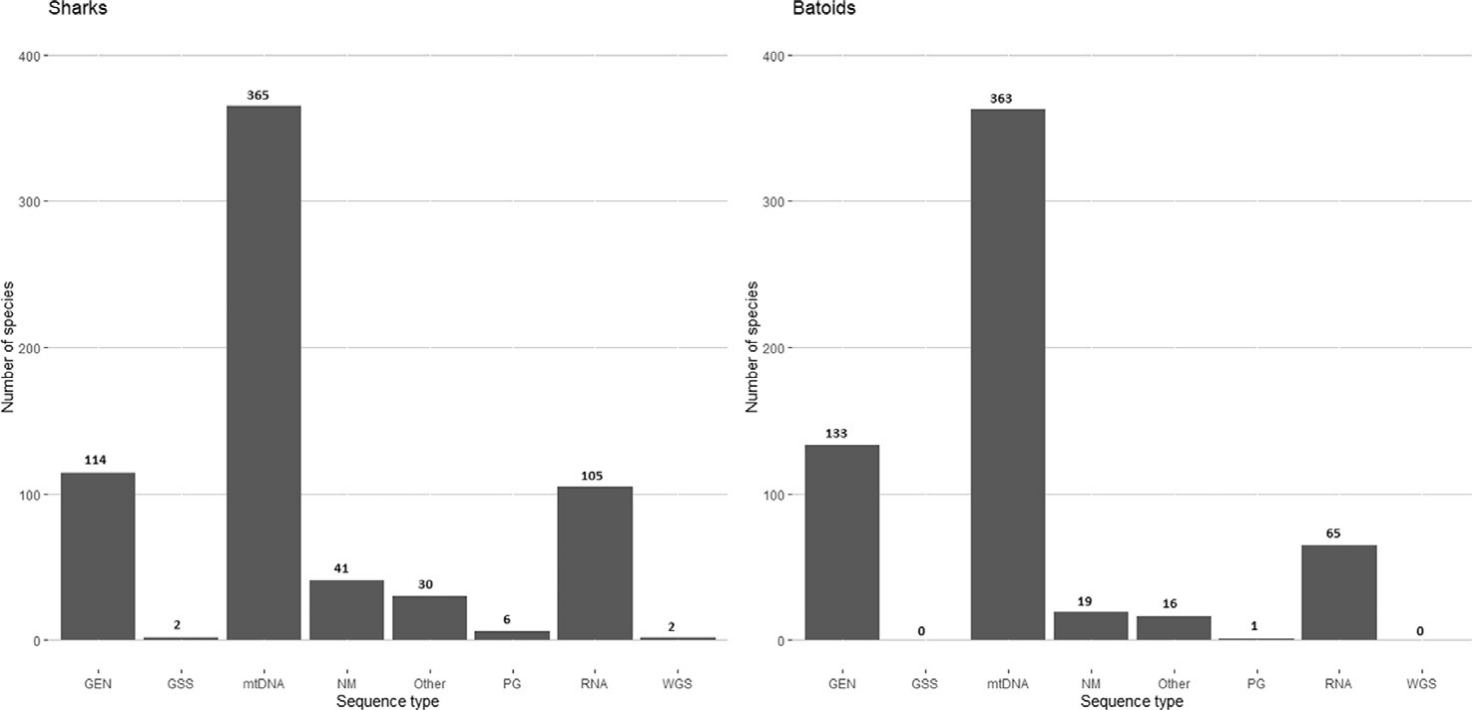
Number of species with records reported on GenBank as a gene sequence (GEN), genomic survey sequence (GSS), mitochondrial DNA (mtDNA), nuclear markers (NM), other types of genomic information, pseudogene (PG), RNA sequences (RNA) or whole genome sequence (WGS).

We assessed sequence data from GenBank to identify the type of information available for each species in each of nine sequence types: 1) gene sequences (GEN), 2) genomic survey sequence (GSS), 3) nuclear marker (NM), 4) mitochondria (mtDNA), 5) pseudogene (PG), 6) RNA sequences (RNA), 7) whole genome sequence (WGS) or 8) other genetic sequences (Other). We found 736 species with records on any of the nine categories. In Supplementary File S1, we list for each species how many records were reported for each sequence type. Fig. 1 summarizes the number of species for which different sequence types were reported for sharks and batoids separately.

For primary biodiversity data, we found 1,017 species of sharks and batoids with occurrence data on the GBIF website between 2000 and April 2020. In Table 3, we show the number of observations for each taxonomic level (e.g., family, genus, species, subspecies, or unknown). For each Elasmobranch order, we display the number of records for: fossils, human observations, living specimens, machine observations, material samples, observations, preserved specimens, and unknown [6]. We filtered the data and counted the total number of observations for each species over the period 2009–2019 for 1) All observation types and 2) only for human observations, machine observations and observations.

**Table 3:** Number of occurrence data points from GBIF (2000–2020) by taxonomic level and observation (obs.) type.

	Taxonomic level						Observation type								
Order	Order	Family	Genus	Species	Sub-species	Unknown	Fossil	Human obs.	Living specimen	Machine obs.	Material sample	Obs.	Preserved specimen	Unknown	Total
Pristiophoriformes	3	0	3	665	0	16	6	579	0	0	37	0	59	6	687
Carcharhiniformes	156	456	2,874	755,514	44	1,766	1,780	177,347	4	573,038	2,255	16	5,556	914	760,810
Lamniformes	22	28	473	13,990	0	192	1,358	10,935	1	1,267	302	3	800	39	14,705
Heterodontiformes	4	8	26	14,448	0	14	6	2,097	0	11,845	444	0	93	15	14,500
Hexanchiformes	2	3	3	5,845	0	19	20	2,508	0	2,238	76	839	169	22	5,872
Myliobatiformes	61	455	1,753	111,455	2	983	732	90,557	21	17,730	1,334	11	3,996	328	114,709
Rajidae	240	2,177	6,019	53,189	5	1,828	78	52,587	0	14	2,233	3	3,622	4921	63,458
Torpediniformes	4	5	191	22,494	0	122	0	21,767	0	0	173	3	553	320	22,816
Rhinopristiformes	6	18	392	22,065	3	119	161	17,671	2	3,703	343	3	641	79	22,603
Squaliformes	148	40	566	32,796	36	1,033	252	27,267	2	136	1,646	4	2,813	2499	34,619
Orectolobiformes	19	43	110	52,283	1	201	30	25,048	0	26,826	257	2	433	61	52,657
Squatiniformes	2	1	24	1,916	0	75	13	1,617	0	0	131	1	228	28	2,018

### 1.2 Conservation status

Conservation status was assessed with IUCN Red List extinction risk assessments and the Alliance for Zero Extinction (AZE) [7]. In the Supplementary File S1 , we list the two batoids in AZE and the current IUCN Red List threat status, the Red List criteria used, the year of assessment, and the species population trend (i.e., declining, stable, increasing, or unknown). For all records, we included a Red List species ID that can be used to merge the data with other IUCN data. In Table 4, we show the number of sharks and batoids that are threatened with extinction (i.e., listed as Vulnerable, Endangered, or Critically Endangered) and break down species in ZIMS, CITES (Appendices I, II, or III), and the Catalogue of Life by threat status. We also indicate the number of species present in those datasets but not assessed by IUCN.

**Table 4:** The number of species in the IUCN Red List database, Species360 Zoological Information Management System (ZIMS), The Convention on International Trade in Endangered Species of Wild Fauna and Flora (CITES), and the Catalogue of Life (CoL). CR: Critically Endangered, EN: Endangered, VU: Vulnerable, NT: Near Threatened, LC: Least Concern, DD: Data Deficient, NA: Not Assessed by IUCN.

				IUCN Red List status						
Taxon		Number of species	Threatened species (%)	CR	EN	VU	NT	LC	DD	NA
Batoids	IUCN	583	123 (21.1%)	26	35	62	55	173	232	-
	ZIMS	97	31 (32.0%)	9	8	14	13	22	21	10
	CITES	46	27 (60.0%)	18	6	3	2	3	14	0
	CoL	689	123 (17.9%)	26	35	62	55	172	230	109

Sharks	IUCN	494	82 (16.6%)	16	22	44	55	170	187	-
	ZIMS	62	12 (19.4%)	3	1	8	18	23	7	2
	CITES	14	14 (100%)	3	5	6	0	0	0	0
	CoL	537	82 (15.3%)	16	22	44	55	169	182	49

### 1.3 Management opportunities

Data for ex-situ management include species holdings and total population size across aquariums in the Species360 network (ZIMS) and whether species are included in a captive breeding program by a regional association. Of the 1,226 species described in the Catalogue of Life, 159 were represented in ZIMS and 18 were managed in regional associations. Of these 18 species, five species were managed only by the European Association of Zoos and Aquaria (EAZA), five species were managed by both the EAZA and the American Association of Zoos and Aquariums (AZA), seven species were managed only by the AZA, and one species was managed by both the AZA and the Aquarium Association Australasia (ZAA).

For in-situ management, we included information on conservation strategies from the IUCN Species Survival Commission (SSC) Shark Specialist Group (SSG) and recovery or action plans under the Australian or Canadian Government, which were the only countries with action plans that were found during the search, either at the family or species level. Additionally, we included national and regional plans both historically and in use from IPOA Sharks [8] which provides an overview of when, and in which countries the action plans are either adopted, drafted, non-official or in progress, though these data do not provide scientific names of species under protection. Further, we included if there is an active recovery plan in place for species and if the habitat lies partly or completely within a protected area according to the IUCN Red List.

### 1.4 Use

This category summarizes information available on the use of the species (e.g., economic, cultural, or medicinal). We included information on international trade in Elasmobranchs between 2000 and 2018 at the species or genus level from the UNEP-WCMC (United Nations Environment Programme World Conservation Monitoring Centre), CITES Trade Database and global catches from the FAO from 2000 to 2017. The dataset in Supplementary File S1 includes CITES information on importing and exporting countries, the origin and source of the species of concern, the purpose of the transaction and quantities, and the terms the species was traded under. We also note the CITES Appendix under which each species is listed. In Table 5 we list terms and purposes of trade. For global FAO catches (2000–2017), we included quantities caught at the species, genus, family and order levels. Catches recorded at the order, the family, or the genus level were divided by the number of species within that taxon to get an overview of which species are possibly caught and at which quantities. We estimated the total catch at the species level as the sum of catches recorded at the order, the family, the genus and the species level for each species. These estimations gave us an overview of which species are possibly caught and at which quantities [9]. Out of the 1,226 species in the Catalogue of Life, six species were listed on CITES Appendix I, 41 on Appendix II, 23 on Appendix III, and 142 were listed on FAO fisheries catches.

**Table 5:** Overview and description of the variables included in the dataset in Supplementary File S1. Descriptions are also listed in Supplementary File S2.

Dataset	Description
CITES	
Time period	2000–2018
Purposes	Commercial (T), personal (P), hunting trophy (H), zoo (Z), scientific (S), circus or traveling exhibition (Q), education (E), law enforcement/judicial/forensic (L), and unknown (NA)
Terms	Carvings, leather products (small), bones, skulls, teeth, derivatives, fins, skin pieces, leather products (large), soup, skins, meat, bodies, live, trophies, specimens, ears, bone pieces, unspecified, medicine, tails, bone carvings, sawfish rostrum, gill plates, jewelry, skeletons, and fingerlings
Sources	Pre-convention (O), wild (W), seized or confiscated (I), unknown (U or NA), captive (C), born in captivity (F), Appendix-I species bred in captivity for commercial purposes (D), and ranched (R)
Units	kg, mg, g, mL, cm^2^ or NA (not available)
Other variables	Importing country, exporting country, country of origin, importer- and exporter-reported quantities
GBIF	
Time period	2000–April 2020 (12 April for the order *Squatiniformes* and 3 April for all other orders)
Record types	Human observation, machine observation, preserved specimen, material sample, fossil specimen, observation, literature, living specimen, and unknown
Taxon levels	Order, family, genus, species, subspecies and unranked
Countries, states, and occurrence statuses	All
FAO	
Time period	2007–2017
Ocean area	Marine areas and inland waters
Land area	Oceania, Africa, Asia, Europe, and the Americas
Data sources included	Catch in tons, data not available, FAO estimate based on available sources or calculations based on specific assumption and null values
FishBase	
Data source	Information Gaps
Variables	Ecology, food, diet, predator, reproduction, spawning, maturity, eggs, hatchling, growth, mortality, length-weight, maximum size, genetics, and photos
Coding	Absent (0) or present (1)
Data source	R Interface to ‘Fishbase’
Information	Body shape, freshwater habitat, brackish water habitat, marine habitat, water zone, migration lifestyle, minimum and maximum depth range, minimum and maximum common depth ranges, longevity in the wild, vulnerability to fishery, length, length type, common length, weight, importance, price category, price reliability, species usage, catching methods – seine, gillnet, cast net, traps, spears, trawls, dredges, lift nets, hook line or other catch methods, used for aquaculture, phylogenetic diversity index, used as bait, used in aquariums, used as gamefish, threat to humans, electrogenic abilities, resilience and trophic level.
Original column names	Yes. We mention the original column names, after extracting the data with the R Interface to Fishbase in the *description* column in the Supplementary File S2.
IUCN Red List	
Version	2020-1
Threat assessments	CR = Critically Endangered, EN = Endangered, Vu = Vulnerable, NT = Near Threatened, LC = Least Concern, DD = Data Deficient, NA = Not assessed
Categories	Criteria, year published, population trend
Version	2020-2
Variables	Country distribution, country code, threats, threat code, stressors, stressors code, trade purpose, trade code, generation length, movement pattern, recovery plan and if protected partly or completely within a protected area
Original column names	Yes. We mention the original column names from the Advanced Search data files “countries”, “threats”, “usetrade” and “all_other_fields” in the *description* column in the Supplementary File S2.

We also included data from the IUCN Red List indicating any purposes of trade for the focal species (e.g human food, sport hunting, medicine, research, jewelry or accessories, chemicals or as pets or display animals). From Fishbase we collated data on catch methods and if the species is used in aquariums, as bait, in aquaculture or as a gamefish.

### 1.5 Conventions and treaties

We collated and standardized the information of seven legal instruments (i.e., international, and/or regional conventions and treaties) protecting or managing Elasmobranchs. In Supplementary File S1, we include if the species is listed in the following international or regional conventions or treaties: CITES (the Convention on International Trade in Endangered Species of Wild Fauna and Flora) both internationally and in the EU, UNCLOS (the United Nations Convention for the Law of the Sea), CMS (the Convention on the Conservation of Migratory Species of Wild Animals), Shark MoU (the Memorandum of Understanding on the Conservation of Migratory Sharks), BERN (the Convention on the Conservation of European Wildlife and Natural Habitats), OSPAR (Protecting and conserving the North-East Atlantic and its resources), and the Barcelona Convention for the Protection of the Marine Environment and the Coastal Region of the Mediterranean.

**Fig. 2:**
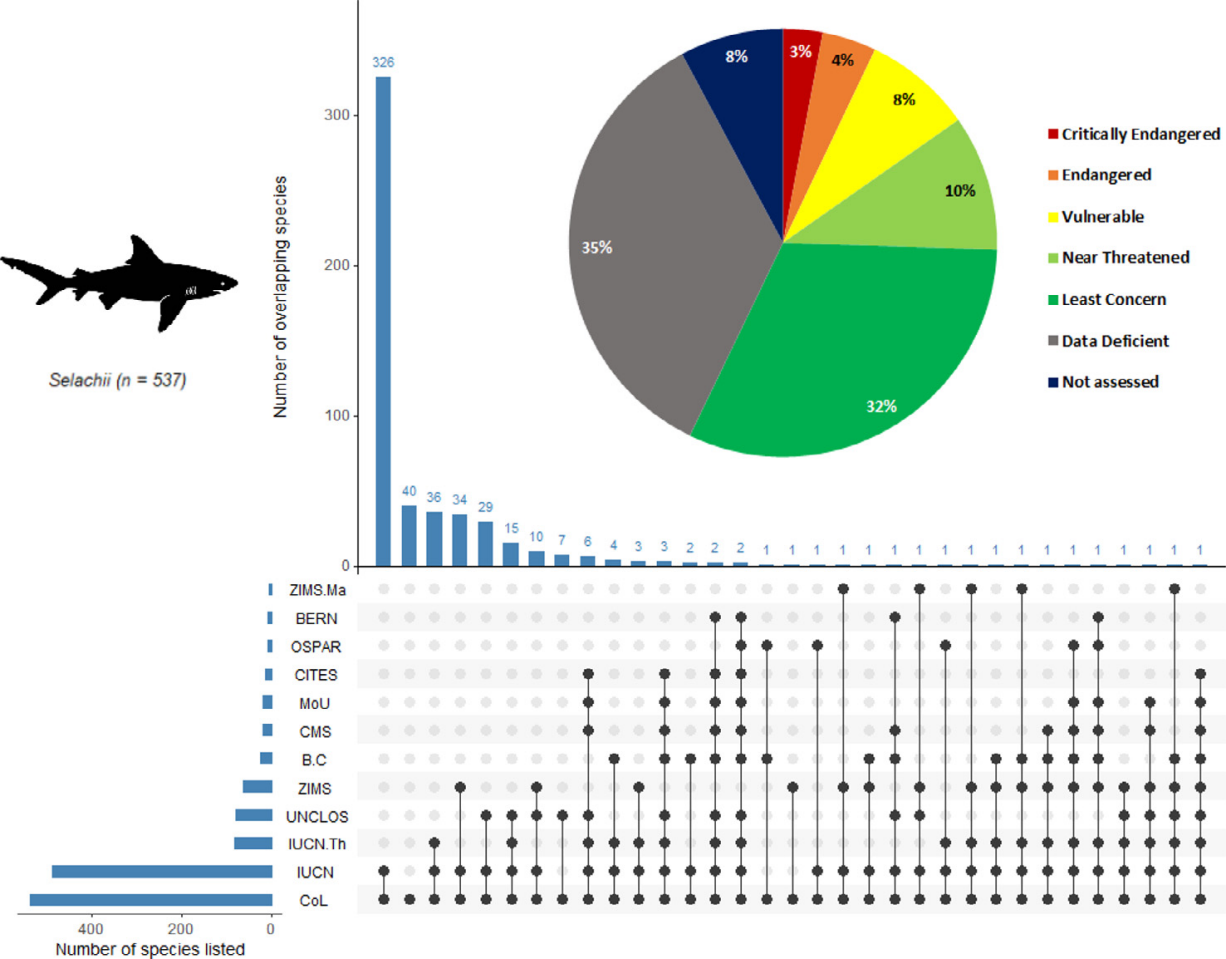
The number of shark species (superorder *Selachii*) in each convention or treaty and their extinction risk status. The pie chart shows IUCN Red List assessments for the 537 extant shark species listed in the Catalogue of Life. In the table below, each dark circle indicates intersections between a convention or treaty. Connected dots represent overlapping species between two or more prioritization schemes. The number of species in each intersection is shown on the upper histogram. The bottom left histogram shows the number of species listed in the convention or treaty. ZIMS. Ma = Species represented in aquariums in the Species360 network and managed in a studbook or breeding program in one or more regional associations. IUCN. Th = Threatened species assessed by the IUCN Red List. Please refer to Table 1 for definitions of all other abbreviations of datasets and conventions or treaties. This plot was generated with the *UpSetR* package (doi:10.1093/bioinformatics/btx364) in the open-source software R [10].

**Fig. 3:**
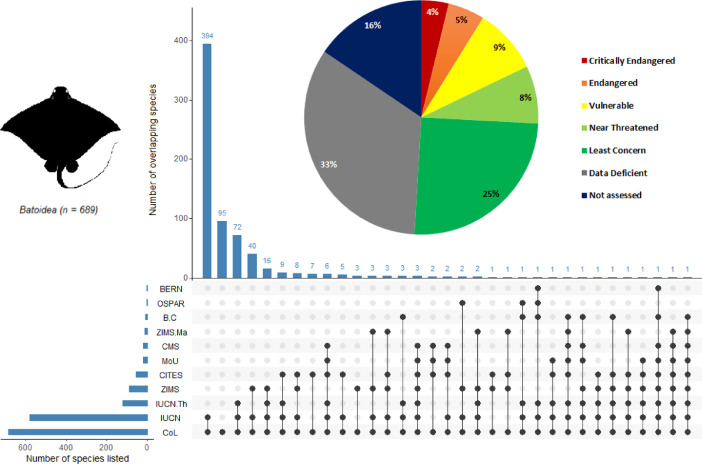
The number of batoids species (superorder *Batoidea*) in each convention or treaty and their extinction risk status. The pie chart shows IUCN Red List assessments for the 689 extant batoid species listed in the Catalogue of Life. In the table below, each dark circle indicates intersections between a convention or treaty. Connected dots represent overlapping species between two or more prioritization schemes. The number of species in each intersection is shown on the upper histogram. The bottom left histogram shows the number of species listed in the convention or treaty. ZIMS. Ma = Species represented in aquariums in the Species360 network and managed in a studbook or breeding program in one or more regional associations: IUCN. Th = Threatened species assessed by the IUCN Red List. Please refer to Table 1 for definitions of all other abbreviations of datasets and conventions or treaties. This plot was generated with the *UpSetR* package (doi:10.1093/bioinformatics/btx364) in the open-source software R [10].

Fig. 2 (sharks) and Fig. 3 (batoids) summarize the overlap between the seven conventions or treaties, the IUCN Red List, and species holdings in aquariums (ZIMS). Nine shark or batoid species (<1%) are not included in the visualizations as their taxonomy did not match across databases (Table 1). We also provide a list of migratory species that can be used to support decision making.

## 2 Experimental Design, Materials, and Methods

To collate data across the 31 open-source repositories, databases, and documents, we standardized the Elasmobranch taxonomy using the Catalogue of Life [1] with the R package *taxize* [10,11], which automatically retrieves species names. When the accepted names were not automatically standardized, we conducted manual checks. Six species on the IUCN Red List were not found in the Catalogue of Life. We retained those in the dataset but list them as NA under the Catalogue of Life (CoL) column. To create the Supplementary File S3, we cross-referenced databases by combining the Catalogue of Life, FishBase, ZIMS, and CITES data into a taxonomic translation table.

Furthermore, we cross-referenced the species taxonomy from CoL with the taxonomy from the World Register of Marine Species (WoRMS) by using the WoRMS Taxon Match Tool available at http://www.marinespecies.org/aphia.php?p=match. We added the scientific name, the taxon status (if taxon is accepted or not according to WoRMS) and the internal taxon ID from WoRMS to the Supplementary File S1. We divided the data by taxonomic orders into sharks: *Hexanchiformes, Squaliformes, Carcharhiniformes, Pristiophoriformes, Heterodontiformes, Lamniformes, Orectolobiformes, Squatiniformes* and batoids: *Rhinopristiformes, Rajiformes, Myliobatiformes, Torpediniformes* [1].

### 2.1 Biological information

We retrieved two types of data from FishBase, 1) we collected the data on biological traits available for the following variables: ecology, food, diet, predators, spawning, maturity, eggs, hatchlings, growth, mortality, length and weight, maximum size, genetics, or photo identification from the Fishbase Information Gaps webpage [5] and, 2) we automatically retrieved data on the species common names, habitat, body shape, water zone, migration, depth ranges and common depth ranges, longevity, length, body weight, resilience to fishery, electrogenic abilities, phylogenetic diversity index, threat to humans, prices, and trophic level using the R Interface ‘rfishbase’ package from the open-source software R [10,12]. To explain words and abbreviations in Supplementary File S2 we used the *List of Symbols and Abbreviations* from Fishbase (https://www.fishbase.in/manual/fishbaselist_of_symbols_and_abbreviation.htm), *the Fishbase System Glossary* (https://www.fishbase.in/Glossary/Glossary.php?q=a&s=index) or the *National Marine Sanctuaries: Fisheries Glossary* - Voices of the Bay (https://sanctuaries.noaa.gov/education/voicesofthebay/glossary.html).

GBIF data were retrieved from GBIF's online database and each Elasmobranch order was downloaded separately (Rhinopristiformes: https://doi.org/10.15468/dl.59ncfb, Rajiformes: https://doi.org/10.15468/dl.un0pej, Torpediniformes: https://doi.org/10.15468/dl.5uuncg, Myliobatiformes: https://doi.org/10.15468/dl.8phc9c, Pristiophoriformes: https://doi.org/10.15468/dl.slawae, Hexaniformes: https://doi.org/10.15468/dl.obnvcs, Heterodontiformes: https://doi.org/10.15468/dl.kfr6u1, Lamniformes: https://doi.org/10.15468/dl.rwwqee, Squaliformes: https://doi.org/10.15468/dl.5yut6b, Carcharhiniformes: https://doi.org/10.15468/dl.54oesv, Orectolobiformes: https://doi.org/10.15468/dl.llxcxj, Squatiniformes: https://doi.org/10.15468/dl.kqmgn3). We indicate which species has been observed between 2000 and April 2020. Additionally, we filtered the data and counted the total number of observations for each species in the time period 2009 and 2019 for 1) All observation types and 2) only for human observations, machine observations and observations [13]. For references and definitions, we refer to the Biodiversity Information Standards Darwin Core quick reference guide [6].

To retrieve genomic information from GenBank, we used the R package *taxize* [11] and obtained a text file with gene descriptions. For simplicity, we classified the data extracted from GenBank into the following sequences 1) gene sequences (GEN), 2) genomic survey sequence (GSS), 3) nuclear marker (NM), 4) mitochondria (mtDNA), 5) pseudogene (PG), 6) RNA sequences (RNA), 7) whole genome sequence (WGS) or 8) other genetic sequences (Other). In Table 6, we list the terms used to search for sequence descriptions in Genbank. We listed all the species with full genome sequences by searching the species list of the Vertebrate Genome Project (VGP; https://vertebrategenomesproject.org/ways-to-help-1). We obtained Evolutionary Distinctiveness (ED) scores for each species from the EDGE of Existence webpage [3].

**Table 6:** Terms used by GenBank and our classification of 10 sequence types.

Sequence type	Terms used by Genbank
Mitochondria	mitochondri
Nuclear marker	microsatellite, single-copy nuclear DNA, marker
Anonymous loci	anonymous nuclear locus, anonymous locus
RNA sequences	miRNA, mRNA, RNA
Pseudogene	pseudogene
Conserved elements	conserved elements
Gene sequences	gene, partial cds, complete cds, complete sequence
Genomic survey sequence	genomic survey sequence
Whole genome sequence	whole genome
Other	All other terms

We gathered data on the species country distribution, generation length range, movement patterns, threats and stressors from the IUCN Red List Advanced Search version 2020-2 using the “country”, “threat” and ”all other fields” files.

### 2.2 Conservation status

We retrieved IUCN Red List assessments from the IUCN Red List of Threatened Species™ version 2020-1 from their website using the taxonomy function, selecting the 12 Elasmobranch orders. We downloaded information from the Alliance for Zero Extinction (AZE) data from the Global AZE map 2018 [7].

### 2.3 Management opportunities

We submitted a request for species holdings data to the Zoological Information Management System (ZIMS) managed by Species360. The species holdings files contain a list of species in Species360-member aquariums globally, the number of adult individuals alive at the time of data extraction and their sex (May 2019).

We obtained the lists of species managed under a breeding program in a studbook from the websites of three regional associations: the European Association of Zoos and Aquaria (EAZA), the American Association of Zoos and Aquariums (AZA), and the Zoo and Aquarium Association Australasia (ZAA).

EAZA reports two types of breeding programs: the European Endangered species Program (EEP) and the European Studbook (ESB). AZA reports on the Species Survival Plan (SSP) as a part of the Marine Fishes Taxon Advisory Group (TAG). ZAA reports on the Australasian Species Management Program (ASMP).

We used the IUCN Species Survival Commission Shark Specialist Group's webpage to gather information on shark and batoid families with a conservation strategy. We assumed that every species under a family with a conservation strategy would have higher in-situ conservation opportunities than those without one. We gathered data on if there is an active recovery plan in place for species and if the habitat lies partly or completely within a protected area from the IUCN Red List Advanced Search version 2020-2 and used the “all other fields” file.

To find action plans at the species level, we used the search engine Google and the keywords: “action plan rays”, “recovery plan rays”, “management plan rays”, “recovery plan sharks”, “action plan sharks”, “management plan sharks”, “management plan Elasmobranchii”, and “action plan Elasmobranchii”. We did not include plans labelled as inactive. The list of species with a recovery, management, or action plan is available in Supplementary File S1. During the search, we found two databases with management plans and action plans at the country level (Australia and Canada). In Table 7, we list all sources used to achieve data on recovery, management, or action plans. Additionally, we included the national and regional plans both historically and in use from other regions of the globe. These were manually retrieved from the interactive map from the FAO International Plan of Action for the Conservation and Management of Sharks [8].

**Table 7:** Sources used to obtain data on species management opportunities, including databases, webpages, and publications.

Authors	Date of access or publication	Title	Link
European Association of Zoos and Aquaria (EAZA)	January 2020	EAZA Ex-situ Programme Overview	https://www.eaza.net/assets/Uploads/CCC/January-2020.pdf
American Association of Zoos and Aquariums (AZA)	February 2020	Animal Program Database	https://ams.aza.org/eweb/DynamicPage.aspx?Site=AZA&WebKey=2b7e3aaf-dc68-49da-ab2e-0dc83ef91a5d&FromSearchControl=Yes
Zoo and Aquarium Association Australasia (ZAA)	February 2020	ZAA-managed species	https://www.zooaquarium.org.au/public/Conservation/Species-Programs/Public/Conservation/Species-Programs.aspx?hkey=c750d8b3-8493-4d92-994c-1bdcc976d23a
Government of Canada	April 2020	Action Plans: Species at Risk Public Registry	Database: https://www.canada.ca/en/environment-climate-change/services/species-risk-public-registry.html Action plan for the Basking Shark: https://species-registry.canada.ca/index-en.html#/documents/205
Government of Australia	April 2020	Species Profile and Threats Database: Recovery plans made or adopted under the EPBC Act	Database: http://www.environment.gov.au/cgi-bin/sprat/public/publicshowallrps.pl Recovery plan for the White Shark: https://www.environment.gov.au/biodiversity/threatened/recovery-plans/recovery-plan-white-shark-carcharodon-carcharias Recovery plan for the Grey Nurse Shark: http://www.environment.gov.au/resource/recovery-plan-grey-nurse-shark-carcharias-taurus Multispecies recovery plan for Sawfishes and River Sharks: http://www.environment.gov.au/biodiversity/threatened/publications/recovery/sawfish-river-sharks-multispecies-recovery-plan
The Shark Trust (Gordon, C.A., Hood, A.R., et al.)	2019	Mediterranean Angel Sharks: Regional Action Plan	https://www.sharktrust.org/Handlers/Download.ashx?IDMF=e8928db3-6d77-455f-93f4-ad4afd1663ac%20
Manta Trust (Ender, I., Stevens, G., Carter, R., Atkins, R. & Copeland, D.)	2018	Conserving Mobulid Rays: A Global Strategy & Action Plan	https://static1.squarespace.com/static/5a196500914e6b09132e911f/t/5c126817562fa7d650d760b3/1544710219568/Global+Strategy+%26+Action+Plan+for+Mobulids_2018_Digital+Download.pdf
IUCN Species Survival Commission	April 2020	IUCN SSC Shark Specialist Group	https://www.iucnssg.org/conservation-strategies.html

### 2.4 Use

Data on international trade was retrieved from the UNEP WCMC CITES Trade Database (2000–2018) for Elasmobranchii. We gathered the data from the FAO (2007–2017) on catches in Oceania, Africa, Asia, Europe, and the Americas for inland waters and marine areas. All marine species within the group “sharks, rays, and chimeras” were selected. Catches recorded at the order, the family, or the genus level were divided by the number of species within that taxon according to CoL, to get an overview of which species are possibly caught and at which quantities. We then estimated the total catch at the species level as the sum of catches recorded at the order, the family, the genus, and the species level for each species [9].

We included the data from the IUCN Red List Advanced Search version 2020-2 indicating the purpose of the trade with the particular species (e.g. human food, sport hunting, medicine, research, jewelry or accessories, chemicals or as pets or display animals), and used the “usetrade” file. To gather data on describing if the species are used: i) in aquariums, ii) as bait, iii) in aquaculture, or iv) as a gamefish and catch methods, we retrieved data from Fishbase using the ‘rfishbase’ package from the open-source software R [10,12].

### 2.5 Conventions and treaties

We obtained names of all Elasmobranch species listed by seven international and regional conventions or treaties: CITES (the Convention on International Trade in Endangered Species of Wild Fauna and Flora), UNCLOS (the United Nations Convention for the Law of the Sea), CMS (the Convention on the Conservation of Migratory Species of Wild Animals), Shark MoU (the Memorandum of Understanding on the Conservation of Migratory Sharks), BERN (the Convention on the Conservation of European Wildlife and Natural Habitats), OSPAR (Protecting and conserving the North-East Atlantic and its resources), and the Barcelona Convention for the Protection of the Marine Environment and the Coastal Region of the Mediterranean.

Information on each convention or treaty was obtained through online databases, legal documents, and official webpages (Table 7). For CMS, we gathered information from press releases following the latest Conference of the Parties (CoP) meeting, since the database had not yet been updated as of February 2020 [2].

## Declaration of Competing Interest

The authors declare that they have no known competing financial interests or personal relationships which have, or could be perceived to have, influenced the work reported in this article.

## Appendix Supplementary materials

Image, application 1

Image, application 2

Image, application 3
